# Supplementary material for: Bioinspired light-driven chloride pump with helical porphyrin channels
Source: Nat Commun. 2024 Jan 27;15:832. doi: 10.1038/s41467-024-45117-1 (PMC10821862; doi:10.1038/s41467-024-45117-1)
Supplement: Supplementary file 1 — Supplementary Information [file 41467_2024_45117_MOESM1_ESM.pdf]

Supplementary information for

“Bioinspired light-driven chloride pump with doping-repaired helical porphyrin channels”

Chao Li[1,2,#], Yi Zhai[1,#], Heming Jiang[3,#], Siqi Li[1], Pengxiang Liu[1], Longcheng Gao[1*], Lei Jiang[1,2]

[1]Laboratory of Bio-Inspired Smart Interfacial Science and Technology of Ministry of Education, School of Chemistry, Beihang University, Beijing 100191, P. R. China

[2]Key Laboratory of Bio-inspired Materials and Interfacial Science, Technical Institute of Physics and Chemistry, Chinese Academy of Sciences, Beijing 100190, P. R. China

[3] Shenzhen Bay Laboratory, Shenzhen 518132, China

[#]These authors contributed equally to this work.

*e-mail: lcgao@buaa.edu.cn

Supplementary Methods

Materials: 5,10,15,20-Tetrakis(4-hydroxyphenyl) porphyrin (TCI, 95%), methyl methacrylate (analytical purity, Beijing Chemical Reagents Co.), and styrene (analytical purity, Beijing Chemical Reagents Co.) were distilled before use. CuBr was synthesized from $CuBr_2$. N,N′,N′,N″,N″-pentamethyldiethylenetriamine (PMDETA, 99.5%, Alfa Aesar), poly (styrene sulfonate) (PSS) (Alfa Aesar), dichloromethane (99.9%, Alfa Aesar) and 2-bromoisobutyryl bromide (97%, Alfa Aesar) were used without treatment. Chlorobenzene and triethylamine (analytical purity, Beijing Chemical Reagents Co.) were treated with powdered CaH_2 and distilled before use.

Synthesis of the star porphyrin initiator: 0.8 g of 5,10,15,20-Tetrakis(4-hydroxyphenyl) porphyrin (1.2 mmol) was added into a 50 mL flask, followed by the addition of 0.35 mL of triethylamine (2.5 mmol) and 20 mL CH_2Cl_2. The mixture was cooled to 0 ^{o}C in the ice-water bath. Then, 0.35 g of 2-bromoisobutyryl bromide (1.5 mmol) was dropwise added into the reactant mixture. After 2 h, the ice-water bath was removed away, and the mixture reacted at room temperature overnight. The postprocessing steps were as follows: First, the mixture was washed with deionized water (DI) 3 times. Then, the accumulated organic solution was dried with anhydrous $MgSO_4$. A clarified organic solvent was accumulated and further concentrated by rotary evaporation. Finally, the crude product was purified by column separation to obtain 1.0 g of purple powder, yielding 66.3%. The ^{1}H NMR result for the star porphyrin initiator (TPP) is shown in Supplementary Fig. 1: δ (ppm, $CDCl_3$): -2.8 (2H, s), 2.28 (24H, s),7.58 (8H, d),8.28 (8H, d),8.90 (8H, s).

Synthesis of the porphyrin-cored star macroinitiator: The macroinitiator was synthesized by the typical atom transfer radical polymerization (ATRP) method. The detailed process was described as follows: Into a 20 mL polymerization tube, 0.2 g of star porphyrin initiator (0.16 mmol), 4.0 g of methyl methacrylate (40 mmol), 0.1 g of PMDETA (0.58 mmol), and 3.0 mL of chlorobenzene were added. The mixture was degassed by three freeze-pump-thaw cycles. And before sealing the tube under vacuum, 0.02 g of CuBr (0.15 mmol) was added into the mixture at the freezing solution state. The reaction tube was placed in an oil bath at 95 °C. After 10 h, the reaction was quenched by dipping the tube in liquid nitrogen, and then the tube was broken. The mixture was diluted with THF, passed through a basic alumina column, and precipitated in methanol two times. The star polymer

p-PMMA was filtered and dried under a vacuum. The product was amaranth solid (3.0 g, monomer conversion 71%). And the chemical structure and molecule weight of the macroinitiator (*p*-PMMA) were confirmed by the ^{1}H NMR spectrum (Supplementary Fig. 2) and the GPC test (Supplementary Fig. 4).

Synthesis of the porphyrin-cored star block copolymer: The star block copolymer was also synthesized by the ATRP method. In a 20 mL polymerization tube, 0.4 g of *p*-PMMA (0.02 mmol), 2.2 g of styrene (22 mmol), 0.017 g of PMDETA (0.10 mmol), and 3.0 mL of chlorobenzene were dissolved. The mixture was degassed by three freeze-pump-thaw cycles. And before sealing the tube under vacuum, 0.003 g CuBr (0.02 mmol) was added into the mixture at the freezing solution state. The reaction tube was placed in an oil bath at 95 °C. After 15 h, the reaction was quenched by dipping the tube in liquid nitrogen and the tube was broken. The mixture was diluted with THF, passed through a basic alumina column, and precipitated in methanol two times. The star block polymer *p*-BCP was filtered and then purified by Soxhlet extraction to remove *p*-PMMA. The dried product was amaranth solid. The chemical structure and molecule weight of the star block copolymer (*p*-BCP) were confirmed by the ^{1}H NMR spectrum (Supplementary Fig. 3) and the GPC test (Supplementary Fig. 4).

Preparation of TPP-doped samples: 0.1 g of *p*-BCP (~0.001 mmol) was mixed with 2.5 mg of TPP (0.002 mmol), 5.0 mg of TPP (0.004 mmol), and 7.5 mg of TPP (0.008 mmol), respectively. The TPP-doped samples were solved by THF to obtain a 1.0 wt% solution. The solution was further treated with 0.22 μm Nylon filters for next-step sample preparation. The doped samples are defined as *p*-BCP@2TPP, *p*-BCP@4TPP, and *p*-BCP@8TPP, respectively.

Fabrication of membrane for electrochemical test: First, the silicon wafer was washed with DI water and ethanol. The 4 wt% PSS solution was spin-coated onto the silicon wafer at a rate of 3000 rpm, functioning as the sacrificial layer. Then, the above prepared 1.0 wt% sample solutions were spin-coated onto the PSS layer at a rate of 3000 rpm, followed by drying under the N_2 atmosphere. The fabricated membranes were thermal-annealed under 200 °C for 24 h. Finally, the membranes were rinsed into DI water to remove the PSS layer and the self-standing porphyrin channel membranes were obtained.

Characterization of the chemical structure and molecule weight: The chemical structure of the porphyrin initiator, macroinitiator (*p*-PMMA), and block polymer (*p*-BCP) were determined by ^{1}H NMR measurements (Bruker AV-500 spectrometer using $CDCl_3$ as solvent). The molecule weight of the polymers was determined by gel permeation chromatography (Waters 2410 instrument equipped with a Waters 2410 Ultraviolet detector), with THF as the eluent at a flow rate of 1.0 mL min^{-1}.

Transmission electron microscopy (TEM): TEM images were recorded using JEM-2100 under s accelerating voltage of 200 kV. The TEM sample was prepared as the following procedure: First, the *p*-BCP sample (2 mg) was solved by 0.4 mL toluene to obtain a 0.5 wt% *p*-BCP solution. Then, the solution was directly dropped onto the copper grid with a carbon membrane. Waiting for its complete evaporation, the sample was thermally annealed under 200 °C for 24 h. To increase the contrast, the continuous phase (PS) was selectively stained with RuO_4 vapor for 20 min.

Energy dispersive X-ray (EDX) mapping: EDX mapping images were recorded with JEM-2100F with an accelerating voltage of 200 kV. The sample solution (0.5 wt% in toluene) was dropped onto a copper grid. Then, the sample was immersed in KCl solution (0.1 M) for 2 hours. Before testing, the sample was rinsed with deionized water to remove the residue salt on the surface.

Scanning electron microscopy (SEM): SEM image was recorded by ZEISS Gemini 300 with a Schottky field emission electron gun at an accelerating voltage of 5 kV. The membrane was transferred onto a silicon wafer. The sample was fractured under a liquid nitrogen condition to expose the cross-section structure.

Grazing-incidence small-angle X-ray scattering (GI-SAXS): GI-SAXS measurements of membranes were carried out with a Xeuss 3.0 system (Xenocs SA, France). The X-ray radiation source with a wavelength of 1.5418 Å (Cu Kα). The scattering signals were received by a charge-coupled device (CCD) detector (Pilatus 300 K, DECTRIS, Swiss) with a resolution of 487 × 619 pixels (pixel size= 172 × 172 μm^2). The distance from the samples to the detector was 1800 mm. The grazing angle was 0.2-0.3°. The samples for GI-SAXS tests were prepared as follows: the above prepared 1 wt% solutions were spin-coated onto the clean silicon wafer at a rate of 3000 rpm. After the completely dry under the N_2 atmosphere, the membranes were thermal-annealed under 200 °C

for 24 h.

Small-angle X-ray scattering (SAXS) and wide-angle X-ray diffraction (WAXD) tests: 2D SAXS and 2D WAXD measurements of samples were also performed on a Xeuss 3.0 system (Xenocs SA, France). The distance from the samples to the detector for SAXS tests was 1800 mm. And the distance from the samples to the detector for WAXD tests was 55 mm. The 2D images were conducted by the Fit2D software to obtain 1D integral curves. The *d*-spacing of the porphyrins and periodic length were calculated by the equation: $d=2\pi/q$. The samples for SAXS and WAXD tests were prepared as follows: The solid doped BCPs were stretched to obtain fiber-like samples at 140 ^oC, above the glass transition temperature of PMMA and PS.

Surface photovoltaic spectroscopy (SPV): The SPV test was carried out with CEL-SPS1000 instrument. The power sample was spin-coated onto the ITO electrode. The illustrated surface voltage under different wavelengths was recorded.

Zeta potential of solid surface: Zeta potentials of prepared membranes were determined with Anton Paar SurPASS. The sample was spin-coated onto a 1 cm×2 cm silicon wafer. The zeta potentials were recorded under different pH solutions.

Circular dichroism (CD) spectra: The CD spectra were tested with the JASCO instrument (J-815). The scanning wavelength ranges from 300 to 600 nm with a rate of 100 $nm\cdot min^{-1}$. The bandwidth was 2.00 nm. The samples for the CD test were prepared as follows: The above prepared 1 wt% solutions were evaporated at room temperature to obtain the doped solid samples. And the samples were thermally annealed and dispersed into dried KBr power with a concentration of 20 $mg\cdot g^{-1}$. Then, the mixture was fully ground and pressed into a semi-transparent sheet with a diameter of 1 cm.

UV-vis absorption spectra: The UV-vis spectra were recorded with Shimadzu UV-3600. The scanning speed was 100 $nm\cdot min^{-1}$. The scanning wavelength range was from 300 nm to 800 nm. And the bandwidth was 4.00 nm. The samples for the UV-vis test were prepared as follows: The above-prepared solutions were diluted to obtain 0.1 wt% solutions. Then, the solutions were directly dropped onto quartz slides. After completely drying, the samples were thermal annealed under 200 ^oC for 24 h.

Fluorescence spectra detection: The fluorescence emission properties of samples were detected

with a Nanolog FL3-2iHR fluorescence spectrometer at room temperature. The excitation wavelength was set as 365 nm. The scanning wavelength range was from 600 to 850 nm. And side entrance slit and exit slit were 2.00 nm. The preparation for the testing samples was similar to that for the UV-vis test.

Infrared thermography: The membrane temperature under light irradiation was in-situ tested by an infrared thermal imager (FOTRIC 220s).

Fourier-transform infrared spectroscopy (FT-IR): FT-IR tests of samples were performed using a Bruker Vertex70, between 400 and 4000 cm^{-1}.

X-ray photoelectron spectroscopy (XPS): The chemical compositions of different crosslinking-degree membrane surfaces were measured by Thermo Scientific K-Alpha under the pressure of 5E-7 mbar with a monochromatic Al Kα X-ray source (hv = 1486.6 eV). All XPS spectra were collected at normal emission and room temperature.

The measurement of light power density: the distance between the light source and the test sample was kept constant, and the light power density irradiated to the membrane is regulated by the power of the light source. The light power density of the light source is measured by a portable light intensity meter. As the energy of the light is dissipated by the electrolyte, the light density irradiated on the membrane is ~4.2 $mW \cdot cm^{-2}$.

Electrochemical measurement: The $I-V$ measurements and energy conversion tests were performed by a Keithley 6487 semiconductor picoammeter (Keithley Instruments) with a pair of Ag/AgCl electrodes. The redox potential was eliminated by the salt bridge, which was prepared according to the literature[1]. The membrane was mounted between a two-compartment conductivity cell. Then, the KCl solution was injected into each cell. The current was recorded with a picoammeter towards different external resistance. The light-responsive ion transport test apparatus is shown in Supplementary Fig. 17, where the helical porphyrin membrane is placed in an electrochemical cell with a quartz window. An external light source can be shone through a quartz window onto the surface of the membrane, and the current is recorded in real-time to evaluate the light-responsive ion transport behavior. The testing membrane was supported by a polyimide membrane with a circular hole diameter of 0.1 mm.

Computational details: All density functional theory (DFT) calculations were conducted with the Gaussian 09 program[2], using the popular functional of B3LYP-D3[3-6]. Geometry optimizations were carried out with the 6-31G(d) basis set for all atoms. All relative energies (corrected with zero point energy) and Gibbs free energies (at 298.15K and 1 atm) are reported in kcal mol^{-1}.

Calculation of the energy conversion efficiency: Under symmetric solution, the light-induced Cl^- migration achieves 56.0 $mW \cdot m^{-2}$ output power density (Fig. 4c). In this case, the membrane resistance is equal to the external resistance. The produced electric power of the whole system is twice the output power. The energy conversion efficiency (η) between light and electricity is calculated to be: $\eta = 2 \times P_1/P_2$, where P_1 and P_2 represent the outpower density of the membrane (56.0 $mW \cdot m^{-2}$) and light power density on the membrane (4.2 $mW \cdot cm^{-2}$=42000 $mW \cdot m^{-2}$). The value is calculated to be ~0.27%.

The determination of simulation model: To simplify the simulation, a single porphyrin channel is taken for analysis. The diameter of the porphyrin channel is equal to the molecular size of the TPP (d=1.6 nm). The nanochannel length (L) is 120 nm based on the cross-section SEM image. If a single electron is excited by light irradiation and migrated to the unillustrated side, the surface charge density on the light-irradicated side could be calculated by: $\varepsilon_1 = e/d^2$. The value is about 0.06 $C \cdot m^{-2}$. Thus, the other side of the porphyrin channel without light irradiation is about -0.06 $C \cdot m^{-2}$. The redistributed surface charge density along the porphyrin channel is set as: ε=0.06 $C \cdot m^{-2}$-0.12 $C \cdot m^{-2} \times X/L$, where X and L represent the X-axial position along the nanochannel and the nanochannel length. To investigate the effect of the redistributed surface charge density on boundary Cl^- concentration, the surface charge density along the porphyrin channel is respectively set as: ① ε=0.01 $C \cdot m^{-2}$-0.02 $C \cdot m^{-2} \times X/L$, ② ε=0.03 $C \cdot m^{-2}$-0.06 $C \cdot m^{-2} \times X/L$, ③ ε=0.06 $C \cdot m^{-2}$-0.12 $C \cdot m^{-2} \times X/L$, ④ ε=0.09 $C \cdot m^{-2}$-0.18 $C \cdot m^{-2} \times X/L$, and ⑤ ε=0.12 $C \cdot m^{-2}$-0.24 $C \cdot m^{-2} \times X/L$.

Supplementary figures

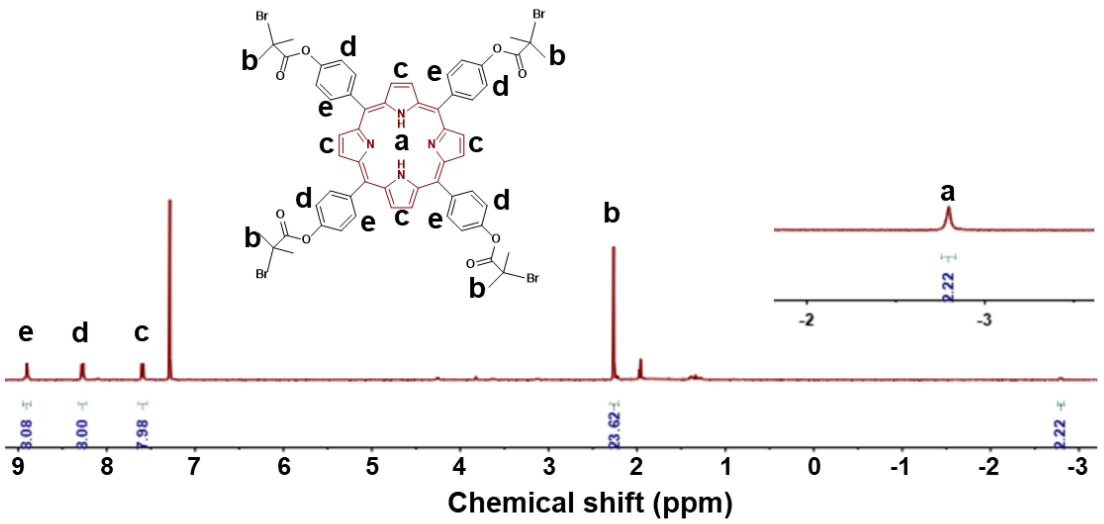

Supplementary Fig. 1 ^{1}H NMR spectrum of porphyrin-cored initiator.

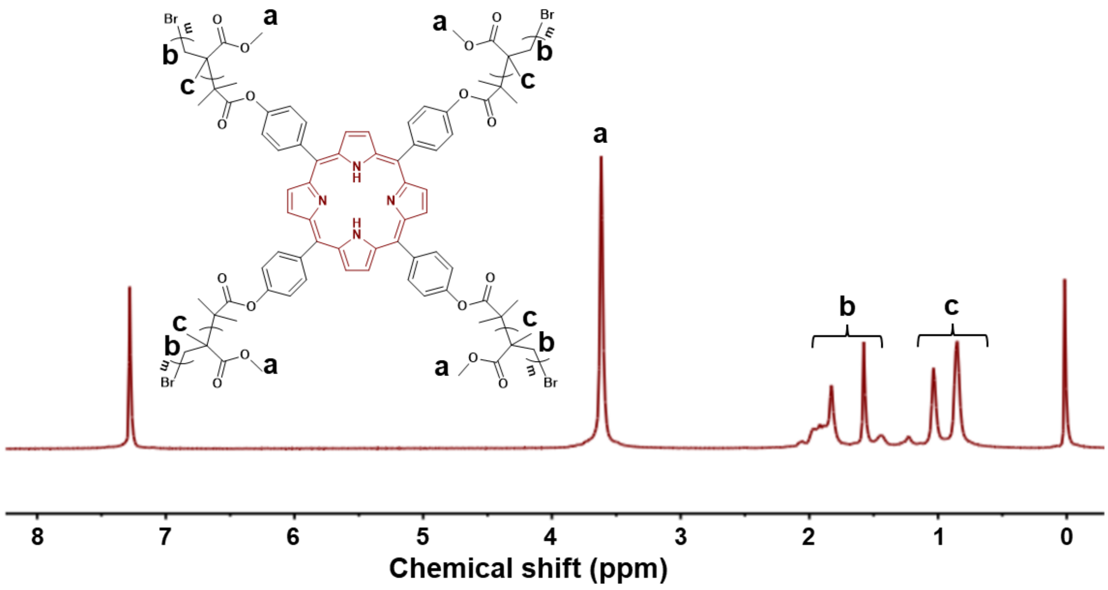

Supplementary Fig. 2 ^{1}H NMR spectrum of porphyrin-cored macroinitiator.

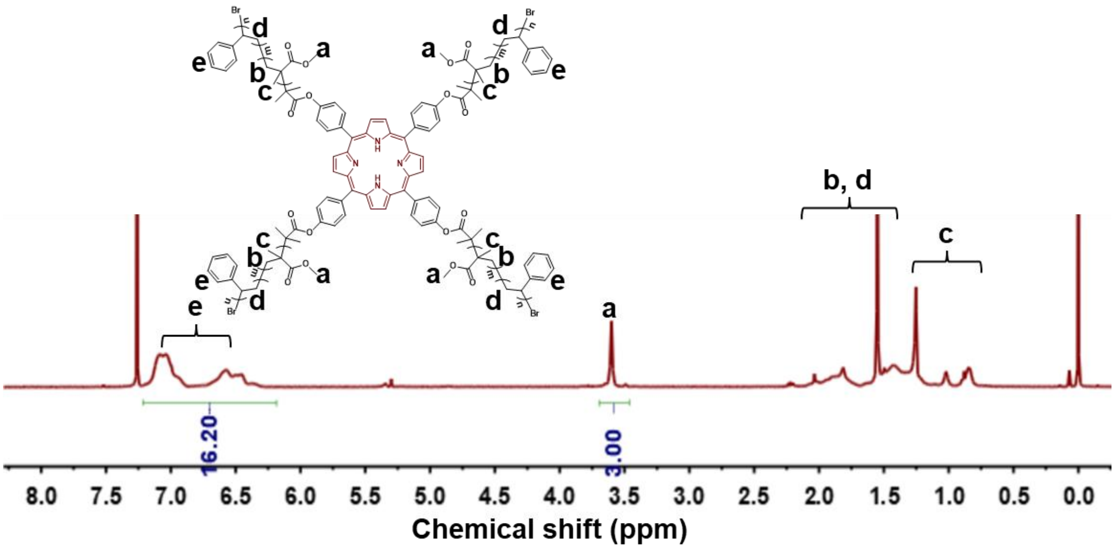

Supplementary Fig. 3 ^{1}H NMR spectrum of the porphyrin-cored block copolymer, indicating the weight fraction of PMMA is ~ 23.6%.

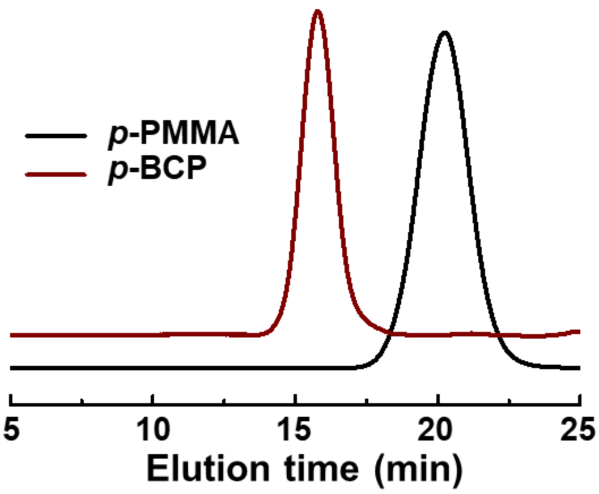

Supplementary Fig. 4 GPC curves of the porphyrin-cored macroinitiator and block copolymer, indicating a narrow distribution of molecular weight.

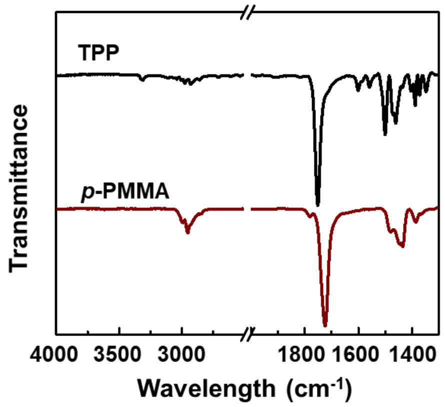

Supplementary Fig. 5 FT-IR spectra of porphyrin-cored initiator (TPP), *p*-PMMA, *p*-BCP, *p*-BCP@2TPP, *p*-BCP@4TPP, and *p*-BCP@8TPP membrane. The signal increases with the doping content of TPP.

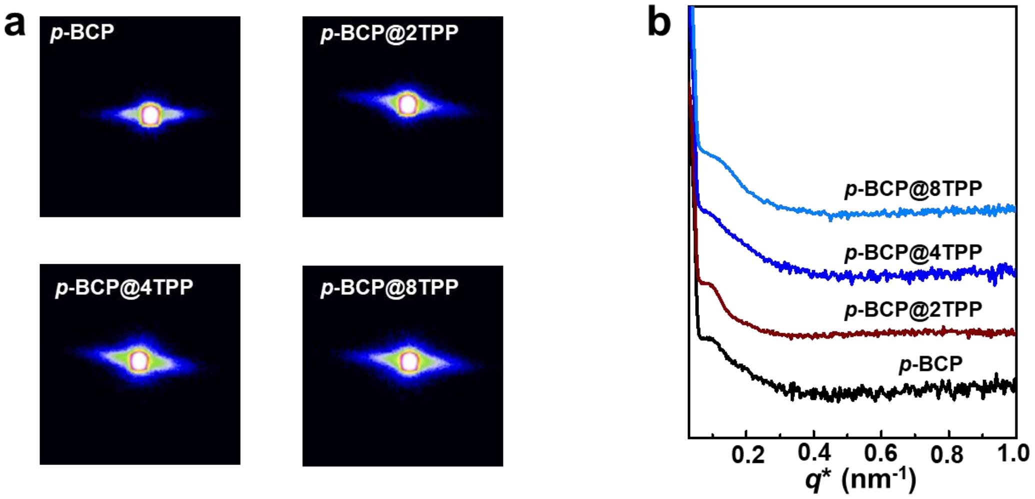

Supplementary Fig. 6 2D small-angle X-ray scattering (SAXS) patterns of *p*-BCP, *p*-BCP@2TPP, *p*-BCP@4TPP, and *p*-BCP@8TPP samples. Two scattering arcs corresponding to the periodic structure are seen on the equator. (b) 1D intensity profiles of 2D SAXS patterns for 2D patterns, showing similar scattering halos with the scattering vectors 0.089 nm^{-1}. The data indicate that the *d*-spacings of PMMA nanocylinders are 70.6 nm.

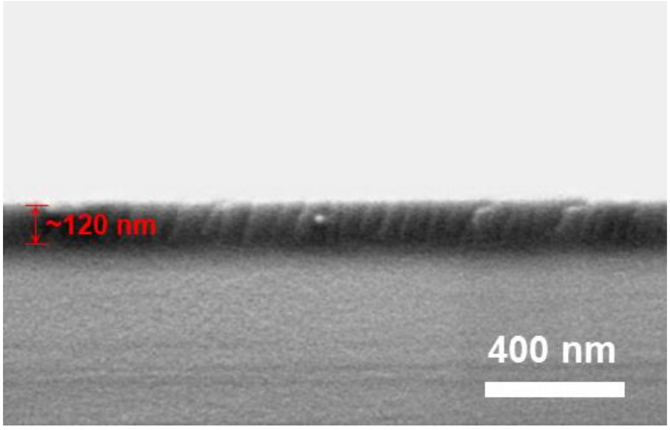

Supplementary Fig. 7. The cross-section SEM image of the *p*-BCP@4TPP membrane, indicating the transmembrane nanocylinder structure (with an average thickness of ~120 nm).

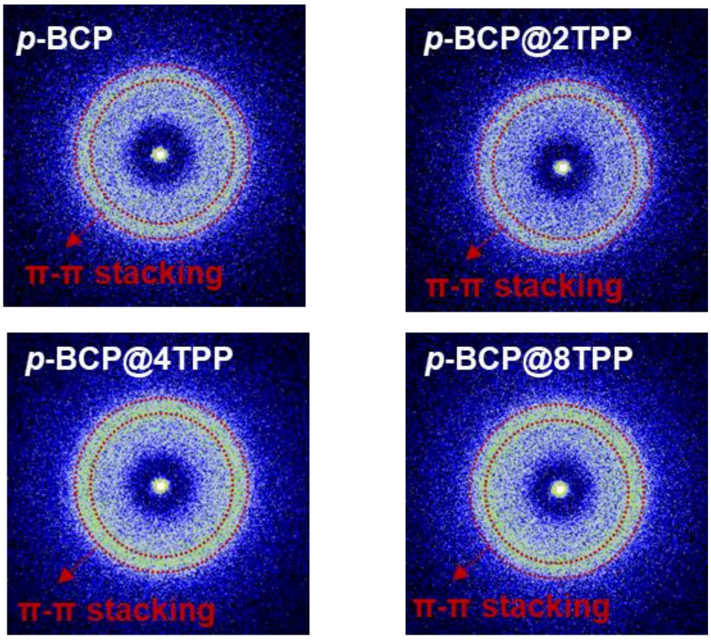

Supplementary Fig. 8 2D WAXD patterns of BCP membranes with different contents of TPP doping. The scattering ring corresponding to the porphyrin π-π stacking is seen from all samples.

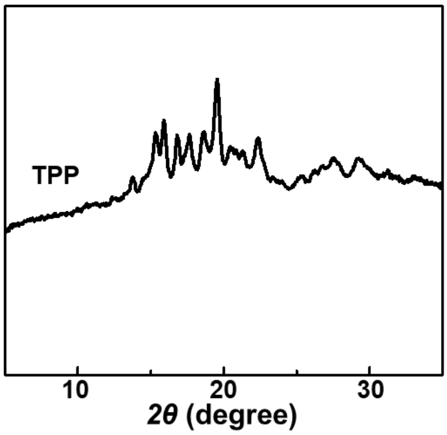

Supplementary Fig. 9 WAXD curves of TPP, showing the diffraction signals for TPP aggregation.

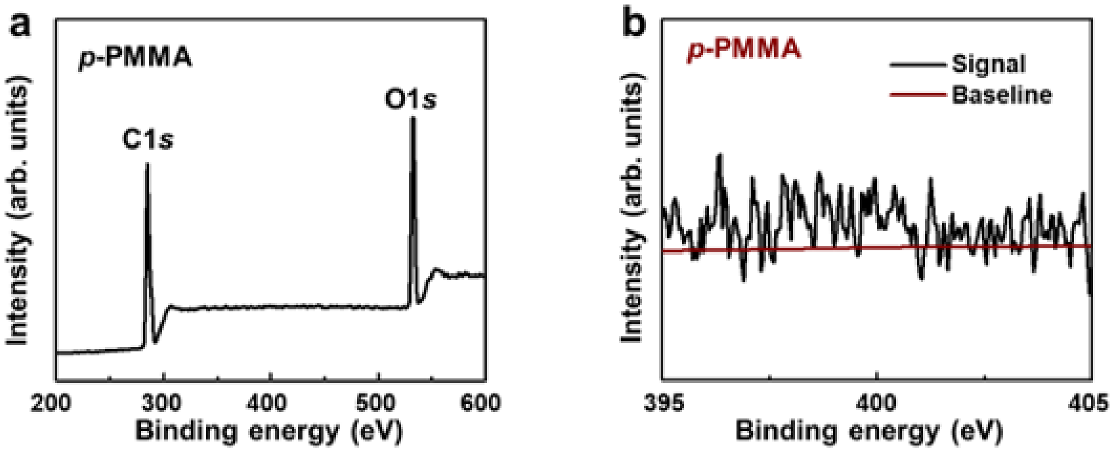

Supplementary Fig. 10 XPS spectra of *p*-BCP membrane. (a) The XPS survey spectra of *p*-PMMA membrane. (b) The scanning N_{1s} XPS spectra of *p*-PMMA membrane. Porphyrins are dispersed in the PMMA chains.

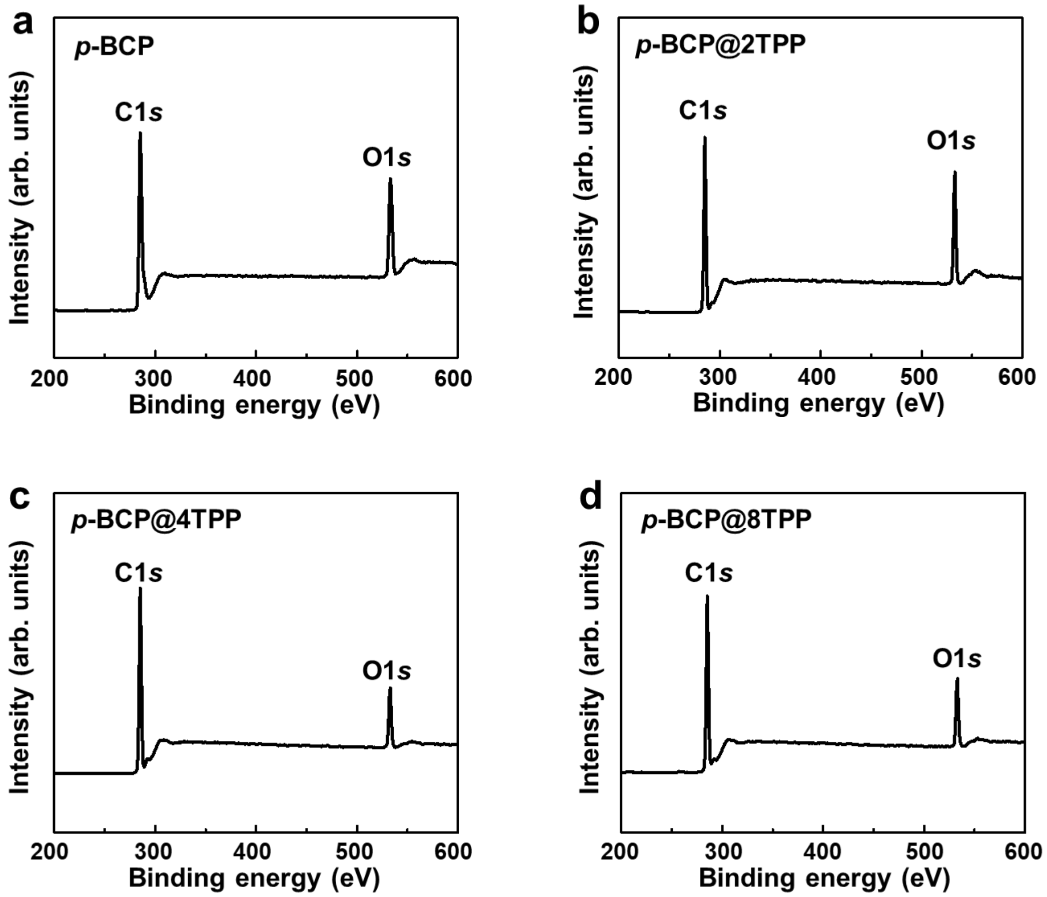

Supplementary Fig. 11 The XPS survey spectra of (a) *p*-BCP membrane, (b) *p*-BCP@2TPP membrane, (c) *p*-BCP@4TPP membrane, and (d) BCP@8TPP membrane.

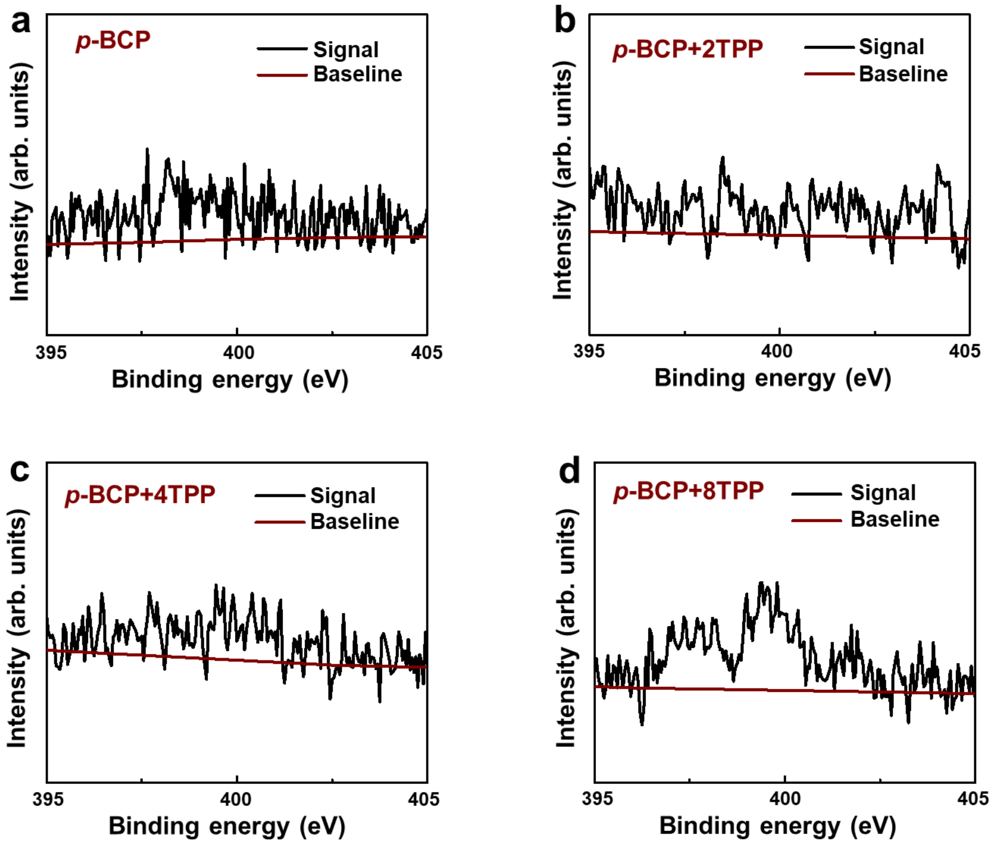

Supplementary Fig. 12 The scanning N_{1s} XPS spectra of (a) *p*-BCP membrane, (b) *p*-BCP@2TPP membrane, (c) *p*-BCP@4TPP membrane, and (d) BCP@8TPP membrane. TPP molecule aggregate in the BCP@8TPP membrane, as the XPS signal of N_{1s} is observed.

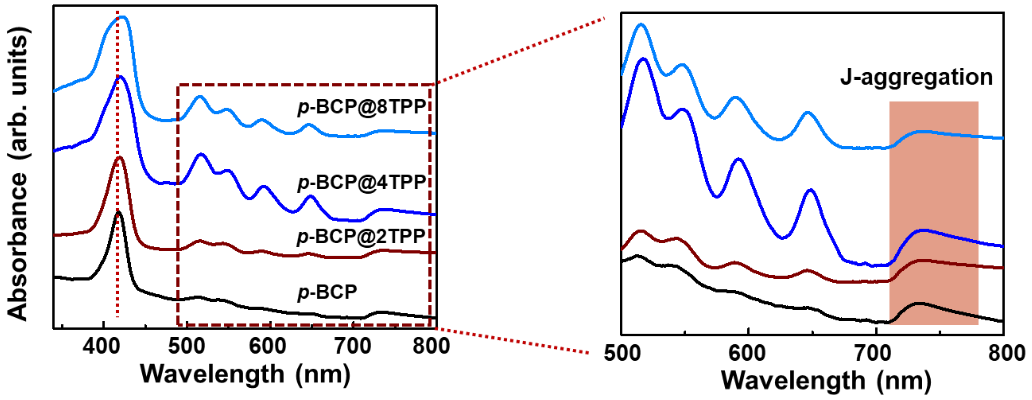

Supplementary Fig. 13 UV–vis absorption spectra of *p*-BCP@*n*TPP samples. The absorption peak at 728 nm indicates the most featured formation of J-aggregation porphyrins.

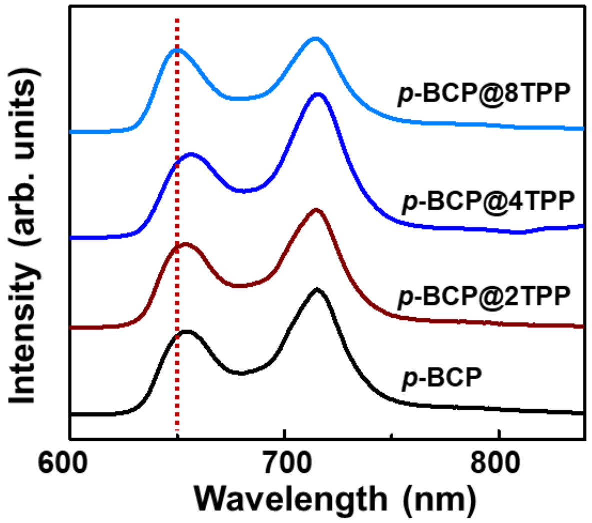

Supplementary Fig. 14 Fluorescence spectra of *p*-BCP@*n*TPP samples. The emission peak of samples at 717 nm corresponds to J-aggregation.

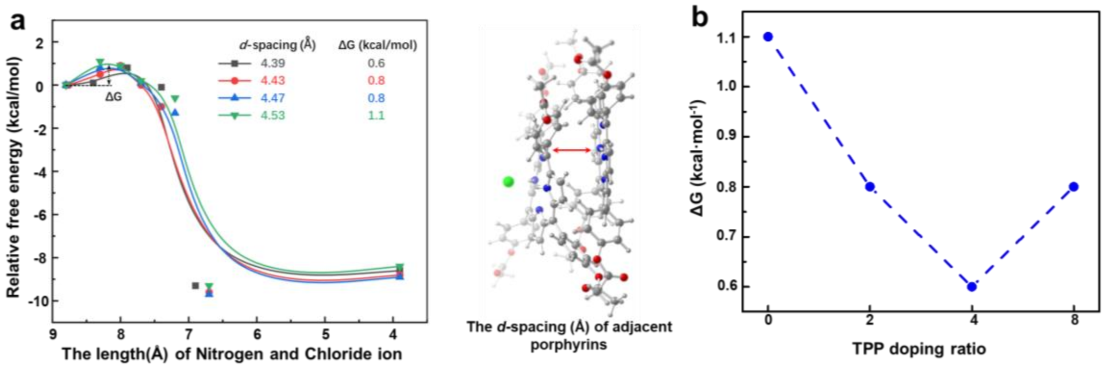

Supplementary Fig. 15 DFT simulation for ion transport with different *d*-spacings. (a) PES section of chloride ion transformation between adjacent porphyrins with different *d*-spacing (Å) along the shortening of Cl^--N distance coordinate, relative free energy is in kcal·mol^{-1}. The different *d*-spacings correspond to different *p*-BCP membranes. (b) The highest relative free energy barrier for Cl^- transformation between the adjacent porphyrins with different distances. *p*-BCP@4TPP shows the lowest relative free energy barrier.

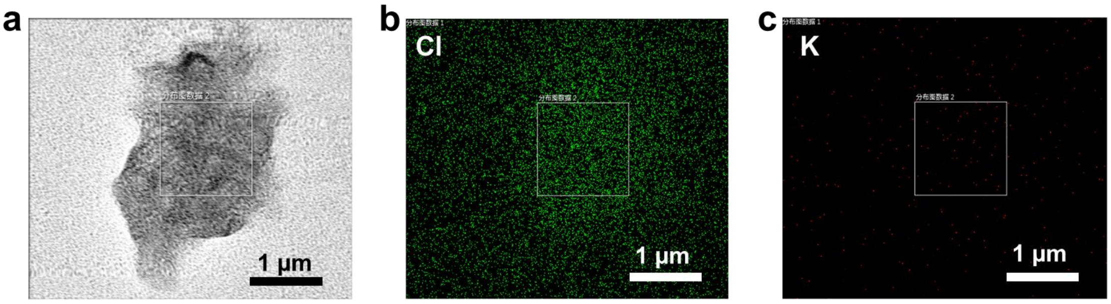

Supplementary Fig. 16 The strong affinity between porphyrin and Cl^-. (a) TEM image of porphyrin aggregates. (b) Energy dispersive X-ray (EDX) mapping of Cl^- with the porphyrin aggregates. (c) EDX mapping of K^+ with the porphyrin aggregates.

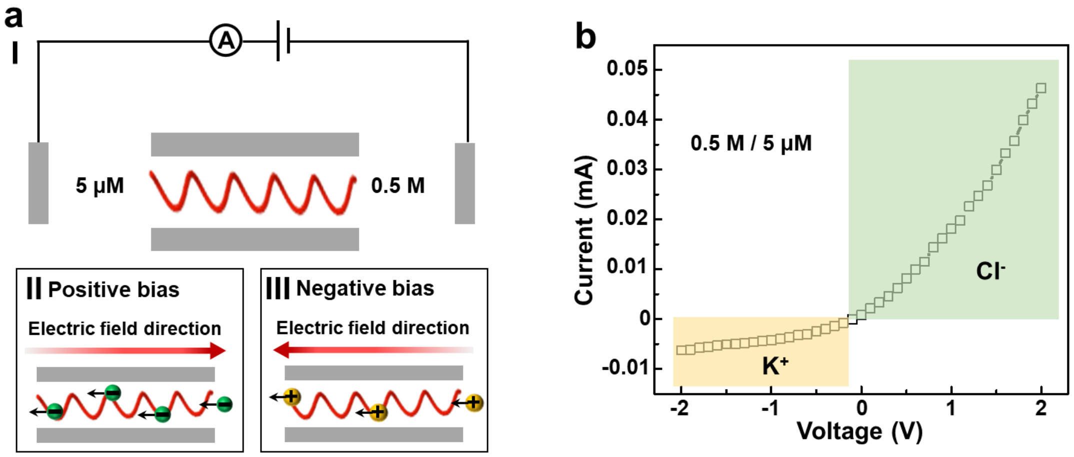

Supplementary Fig. 17 The Cl^- selectivity of the porphyrin channel membrane. (a) The electrochemical testing condition for the I-V curve under an ultra-asymmetric concentration gradient. The positive electrode is placed on the 0.5 M concentration gradient. (b) I-V curve of the *p*-BCP@4TPP membrane under an ultra-asymmetric concentration gradient (0.5 M / 5 μM). The current at positive bias is higher that that of negative bias, indicating that the membrane shows Cl^- selectivity.

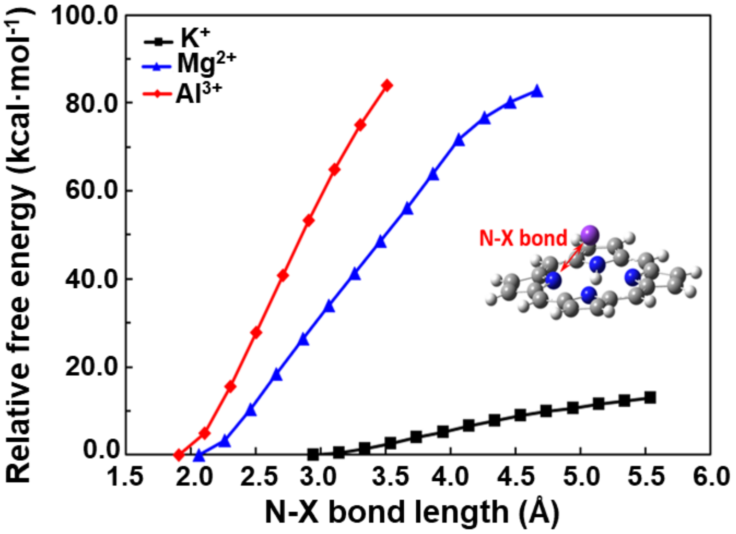

Supplementary Fig. 18 The relative free energy barriers of scan results for different cations (scanning the N-X (X=K^+, Mg^{2+} or Al^{3+}) bond in red color). The relative free energy barriers are much higher than that of Cl^- (~0.6 kcal·mol^{-1} shown in Supplementary Fig. 15).

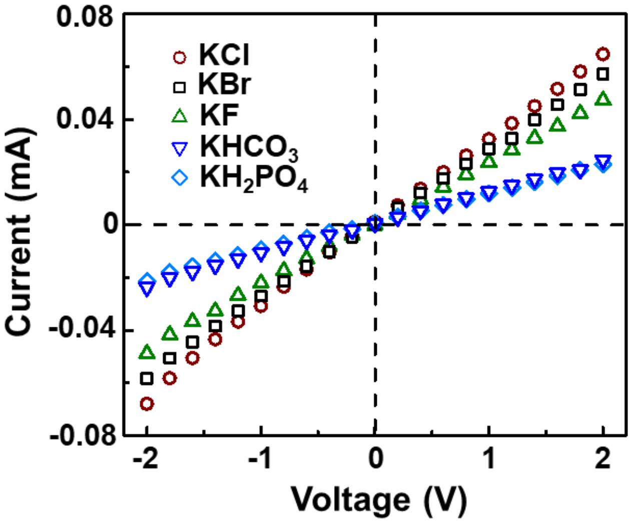

Supplementary Fig. 19 Current-voltage curves of the *p*-BCP@4TPP membrane with different electrolytes, exhibiting the highest conductance for Cl^- transport.

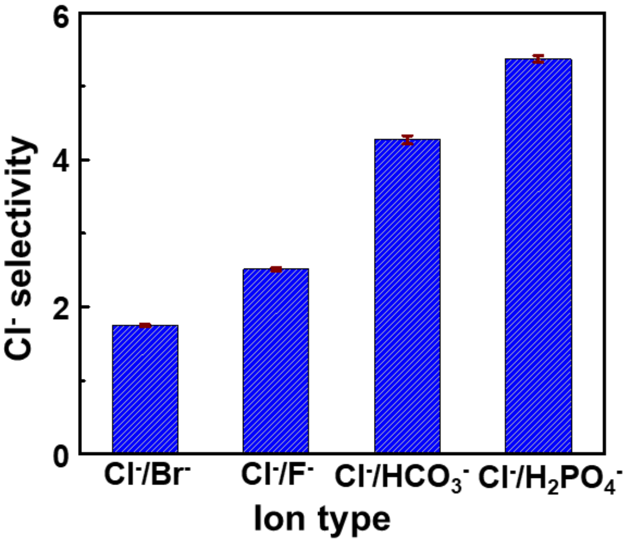

Supplementary Fig. 20 Cl^- selectivity toward Br^-, F^-, HCO_3^-, and $H_2PO_4^-$ for the *p*-BCP@4TPP membrane.

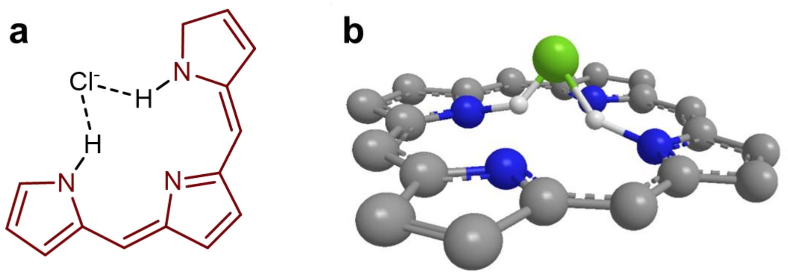

Supplementary Fig. 21 The molecular structure of the hydrogen bond. (a) The hydrogen bond between Cl^- and porphyrin (partial structure). (b) Schematic graph of porphyrin species interacting with chloride anion. The green atoms represent chloride anion.

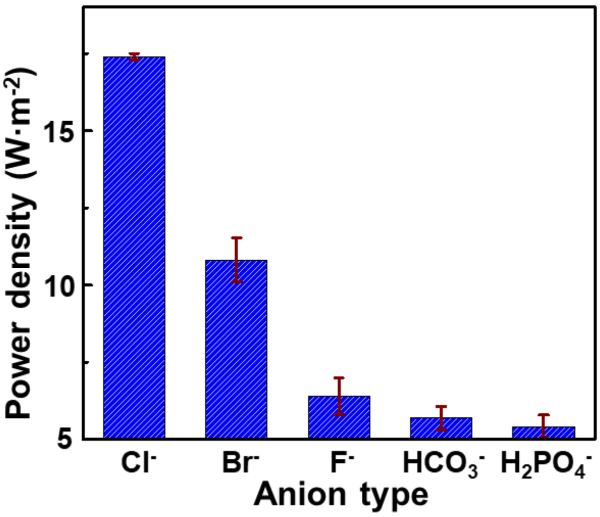

Supplementary Fig. 22 Maximum output power densities of the *p*-BCP@4TPP membrane with different anionic electrolytes, showing the highest power density for Cl^-.

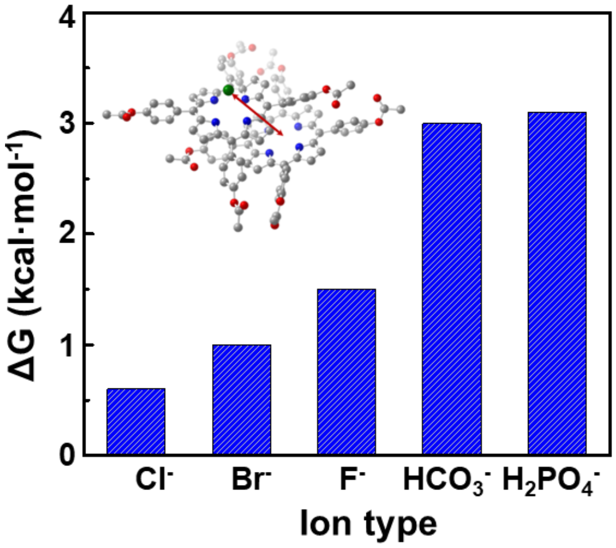

Supplementary Fig. 23 The highest relative free energy barrier for anion transformation, showing the lowest relative free energy barrier for Cl^- transformation.

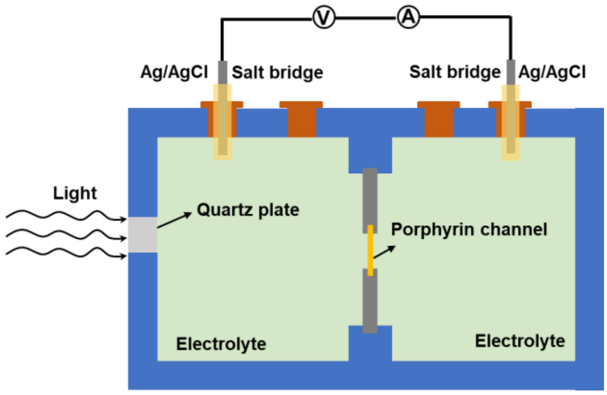

Supplementary Fig. 24 Illustrated diagram of the light-driven ion transport test. The redox potential and redox current are eliminated by the salt bridge. The positive diffusion potential and negative diffusion current could be directly obtained by the open voltage and short-circuit current.

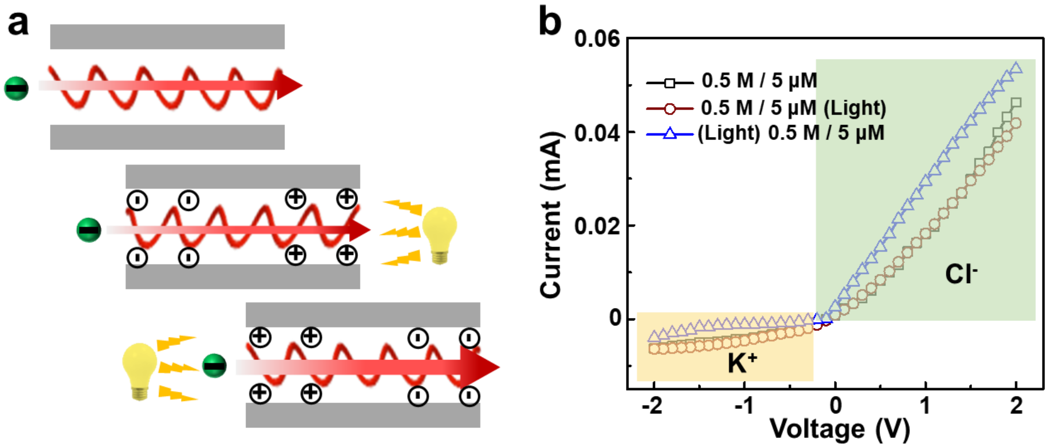

Supplementary Fig. 25 The ion selectivity of the membrane toward light irradiation. (a) The illustrated diagram of the ion transport without and with light irradiation. (b) I-V curves of the *p*-BCP@4TPP membrane under an extremely asymmetric concentration gradient.

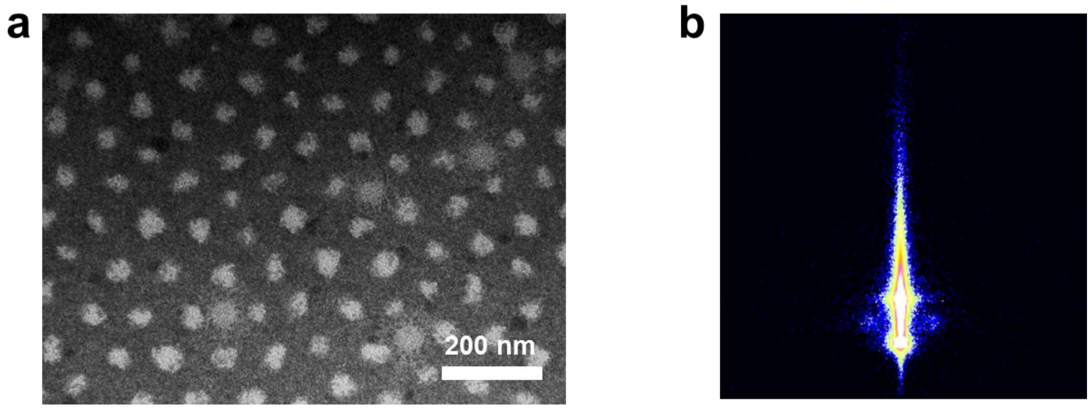

Supplementary Fig. 26 The nanostructure stability of porphyrin channel membrane toward light stimulation. (a) TEM image of *p*-BCP@4TPP membrane after light irradiation for 1 hour. (b) 2D GI-SAXS patterns of *p*-BCP@4TPP membrane after light irradiation for 1 hour.

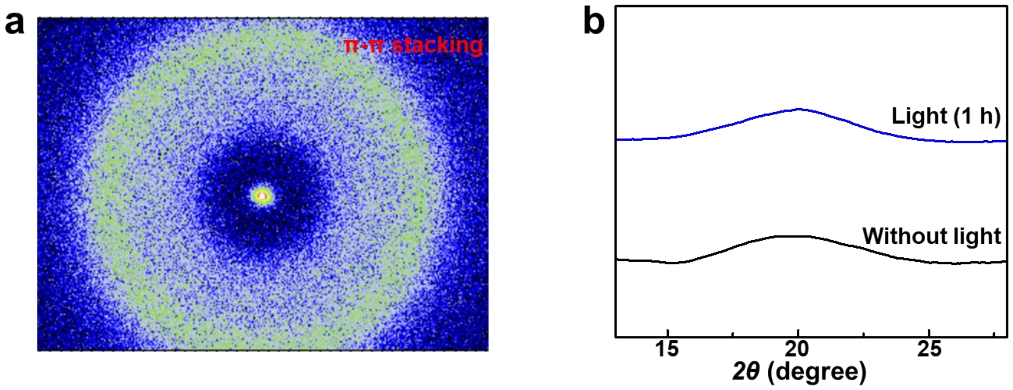

Supplementary Fig. 27 The porphyrin aggregate stability toward light stimulation. (a) 2D WAXD pattern of *p*-BCP@4TPP membrane after light irradiation for 1 hour. (b) The WAXD curves of *p*-BCP@4TPP membrane before and after light irradiation.

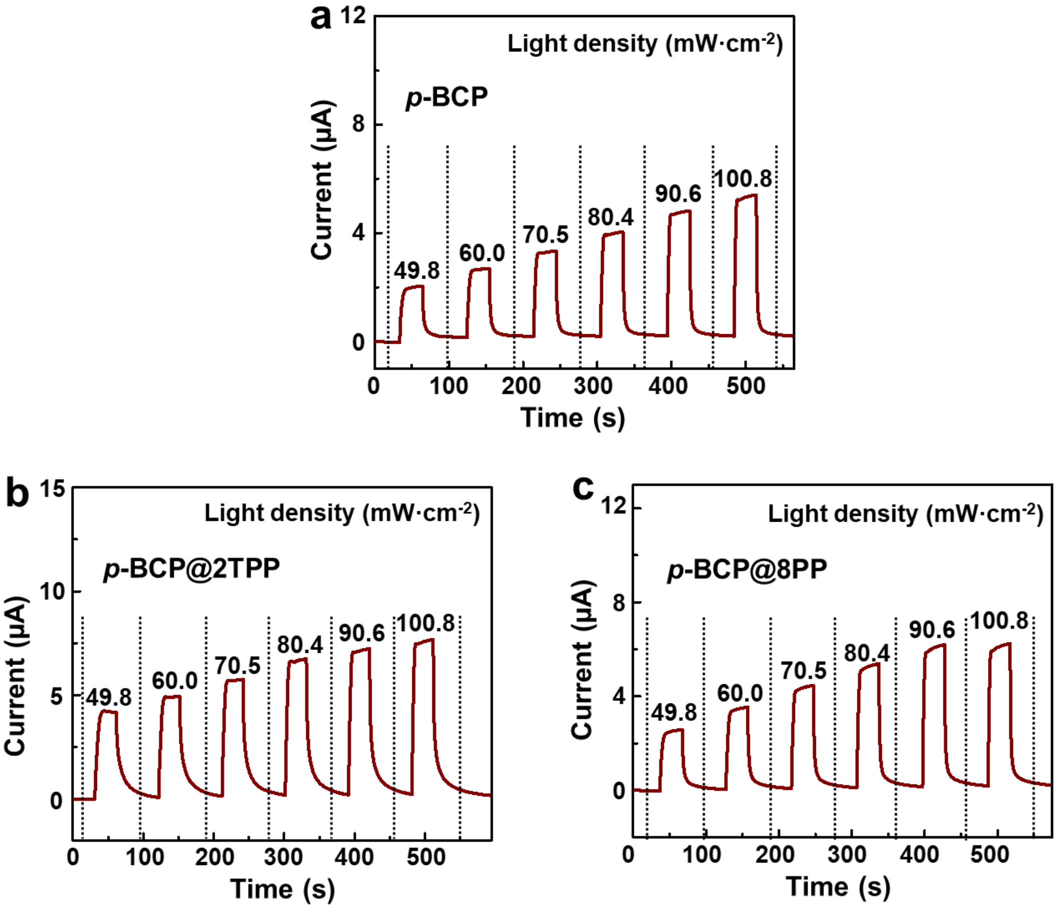

Supplementary Fig. 28 The time-dependent photocurrent curve of the (a) *p*-BCP, (b) *p*-BCP@2TPP, and (c) *p*-BCP@8TPP membranes under a + 1.0 V bias with different light densities. All samples exhibit increased photocurrent value with the increased light density.

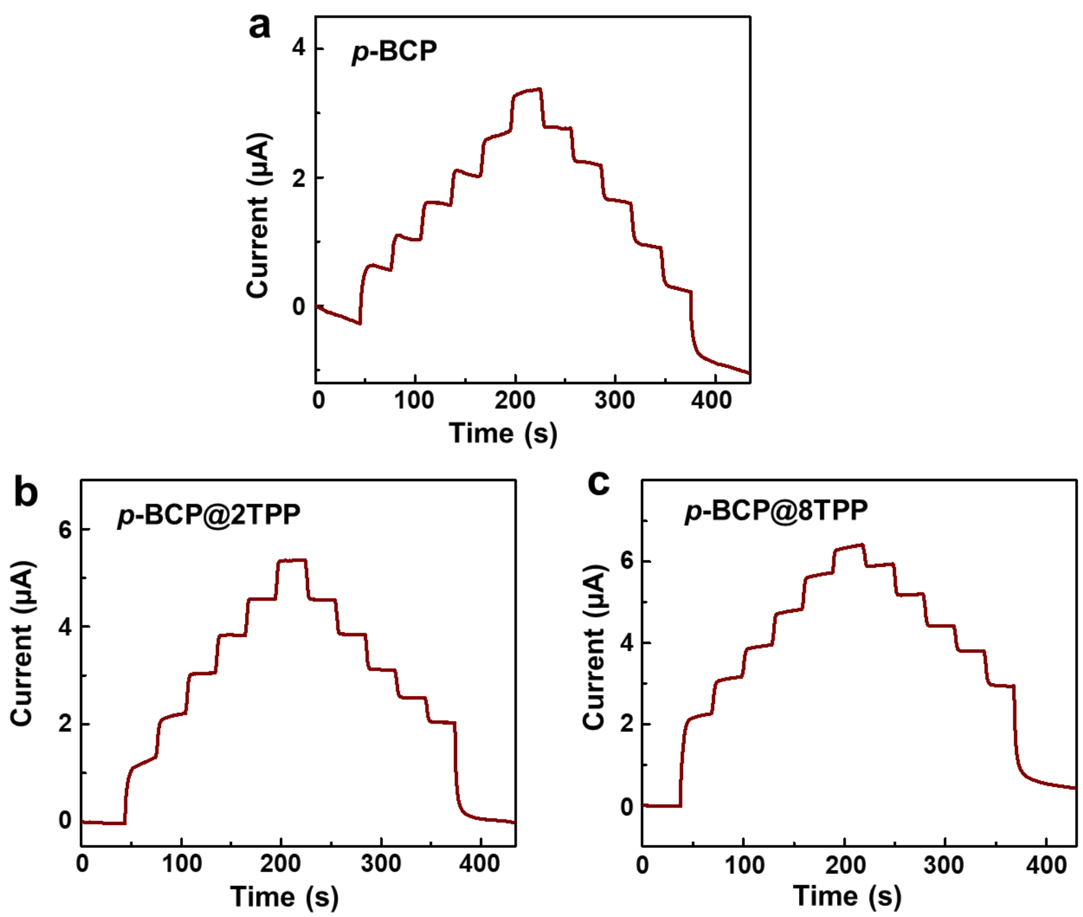

Supplementary Fig. 29 The time-dependent photocurrent curve of(a) *p*-BCP, (b) *p*-BCP@2TPP, and (c) *p*-BCP@8TPP membranes under a + 1.0 V bias with different light densities. All samples exhibit excellent reversible light-responsive ion transport.

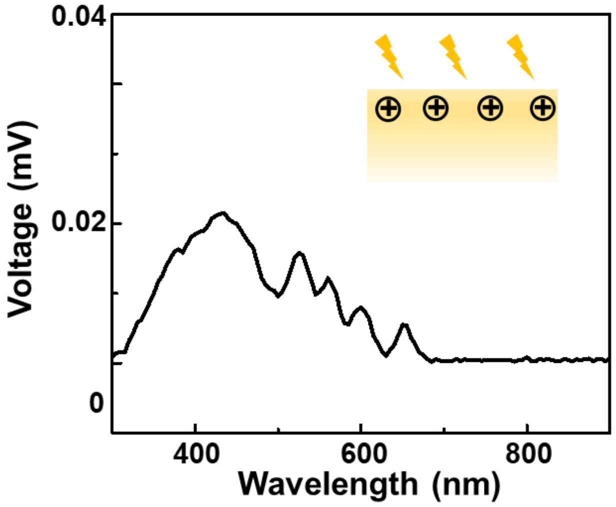

Supplementary Fig. 30 SPV spectra of *p*-BCP@4TPP membrane, indicating that light induced positive charge generation.

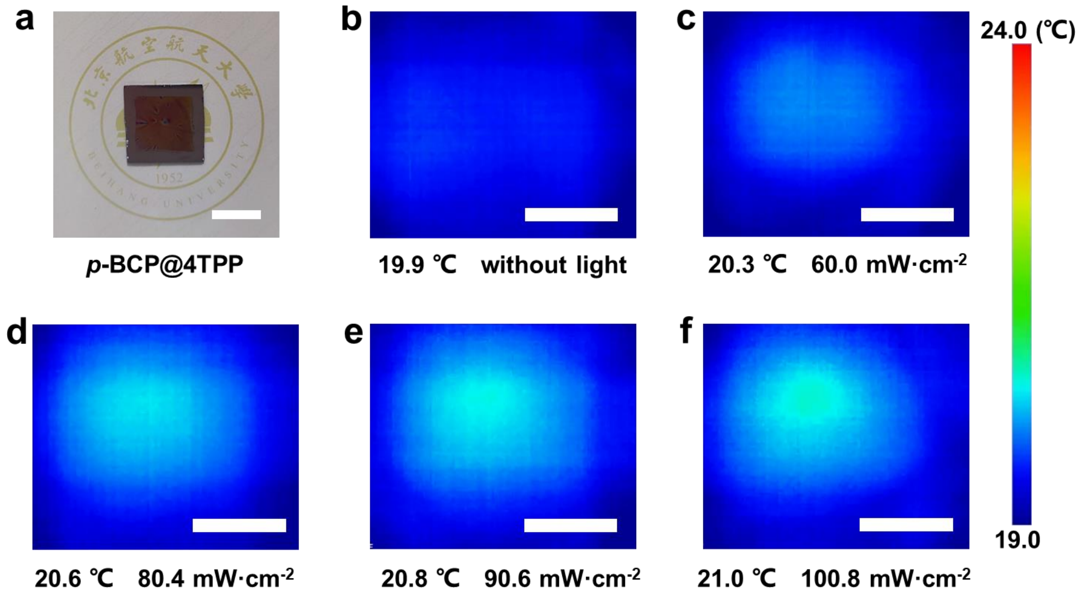

Supplementary Fig. 31 Infrared images of *p*-BCP@4TPP membrane under different light densities. (a) Photograph of the *p*-BCP@4TPP membrane (Scale bar: 1 cm). The infrared thermography of *p*-BCP@4TPP membrane (b) without light, and exposed under UV light with density of (c) 60.0 mW·cm^{-2}, (d) 80.4 mW·cm^{-2}, (e) 90.6 mW·cm^{-2}, and (f) 100.8 mW·cm^{-2} (Scale bar: 0.5 cm).

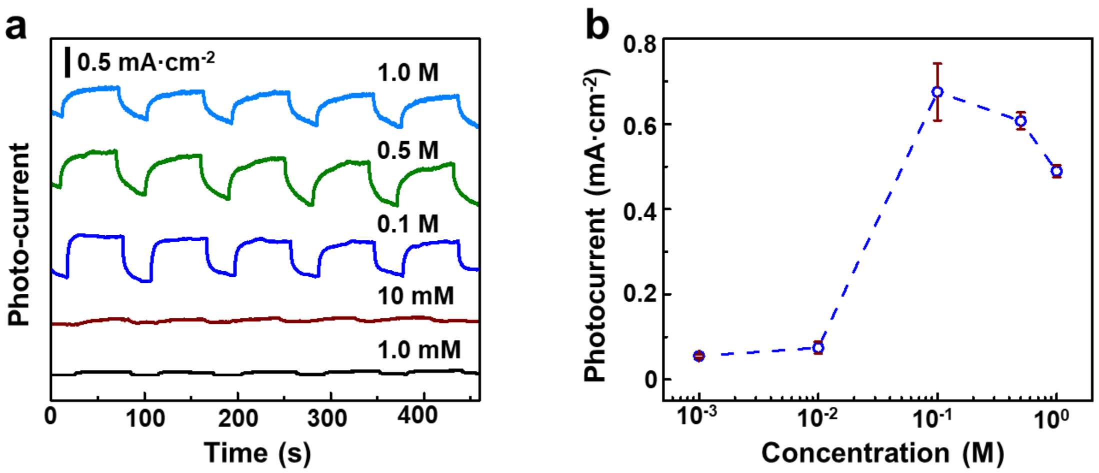

Supplementary Fig. 32 (a) The time-dependent photocurrent densities of the *p*-BCP@4TPP membrane in different-concentration electrolytes with light irradiation (100.8 mW·cm^{-2}). (b) The photocurrent densities of the *p*-BCP@4TPP membrane as a function of the electrolyte concentration, indicating the optimal concentration (0.1 M). Error bars represent standard deviation (n=3).

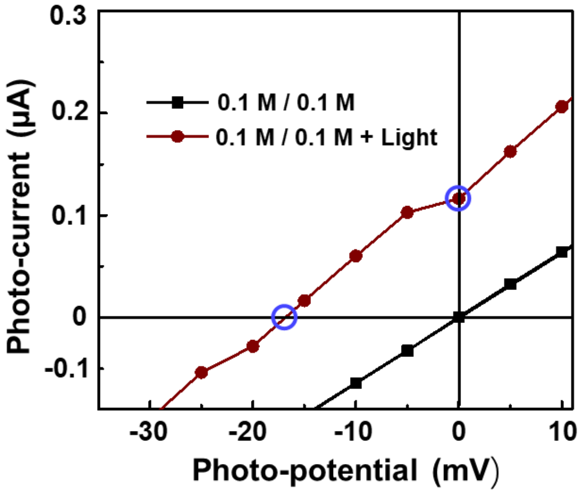

Supplementary Fig. 33 Current-voltage curve of the *p*-BCP@4TPP membrane in symmetric electrolyte with and without light irradiation. The diffusion potential and diffusion current are generated by the light-driven Cl^- selective transport.

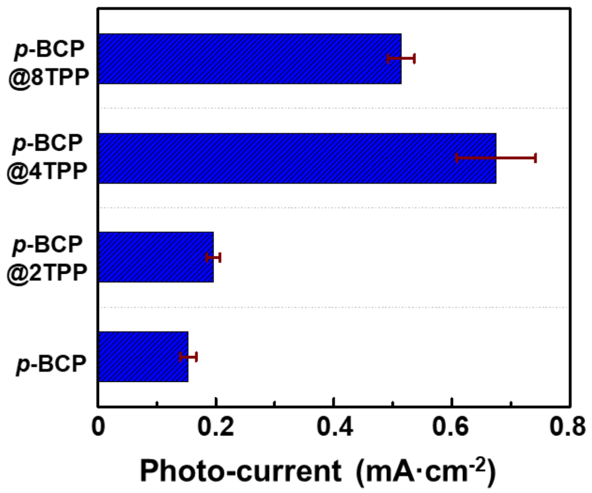

Supplementary Fig. 34 The comparison of light-driven ion currents for *p*-BCP@*n*TPP membranes at a light density of 100.8 mW·cm^{-2}. Error bars represent standard deviation (n=3).

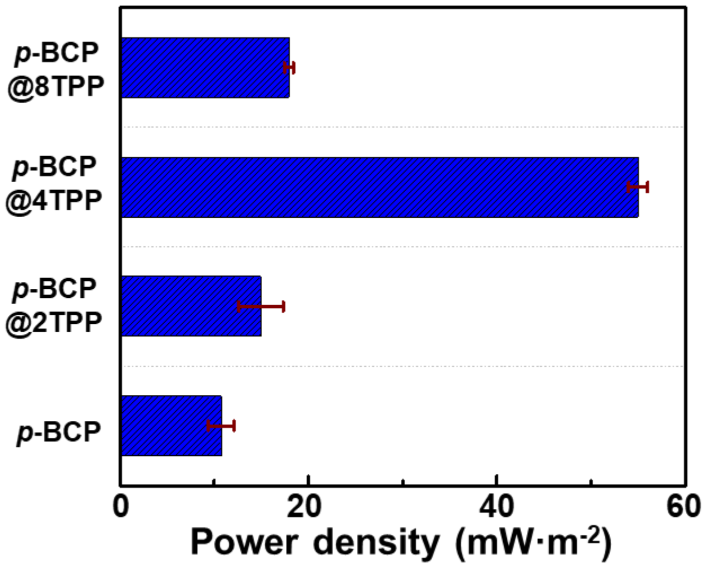

Supplementary Fig. 35 Maximum output power densities of the *p*-BCP@*n*TPP membranes, showing the highest power density for the *p*-BCP@4TPP membranes. Error bars represent standard deviation (n=3).

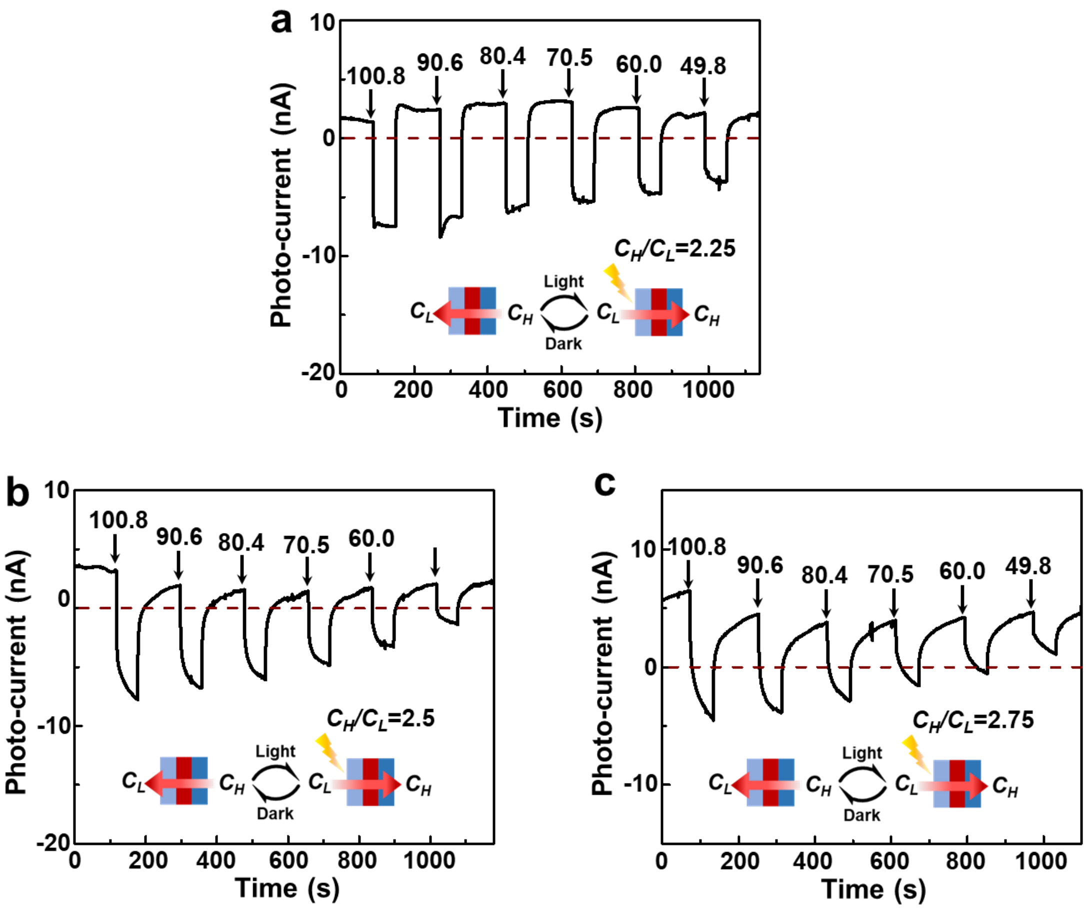

Supplementary Fig. 36 The time-dependent photocurrent of the *p*-BCP@4TPP membrane under different asymmetric concentration gradients (a) C_H/C_L=2.25, (b) C_H/C_L=2.5, and (c) C_H/C_L=2.75 with different light densities. The inversed current indicates the inversed ion transport directions.

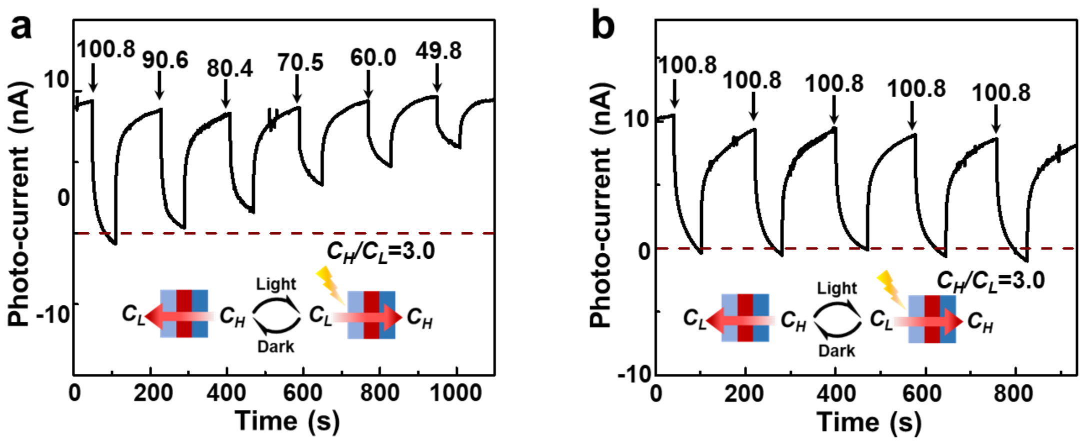

Supplementary Fig. 37 (a)The time-dependent photocurrent of the *p*-BCP@4TPP membrane under a 3-fold concentration gradient (C_H/C_L=3.0) with different light densities. (b) The time-dependent photocurrent cycle of the *p*-BCP@4TPP membrane with a light density of 100.8 mW·cm^{-2}.

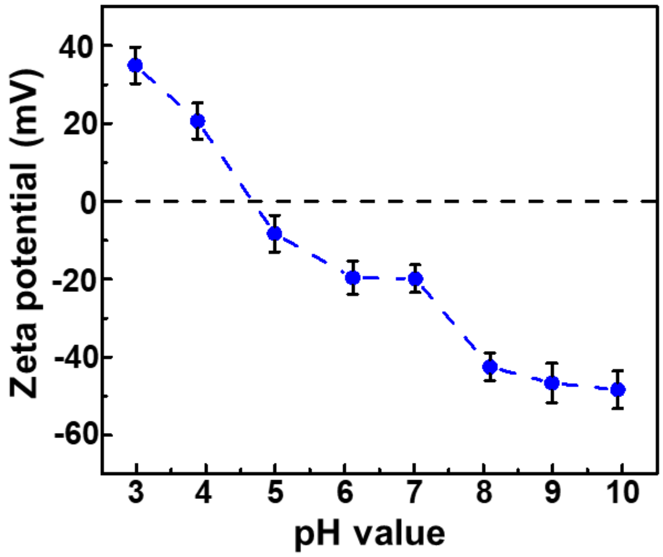

Supplementary Fig. 38 Zeta potential of the porphyrin membrane, showing positive membrane surface under acidic condition. The porphyrin attracts Cl^- above 4.7, showing a negative zeta potential. Error bars represent standard deviation (n=3).

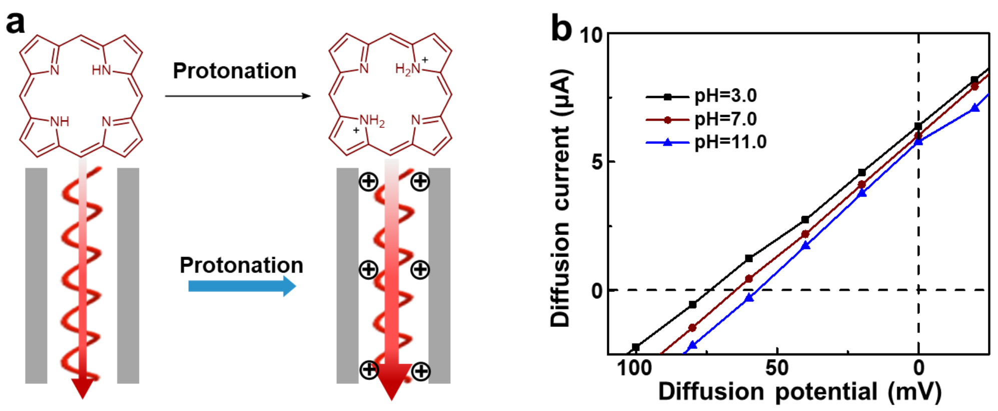

Supplementary Fig. 39 The Cl^- selectivity toward pH value. (a) The illustrated diagram of the protonated porphyrin under acidic conditions. (b) The I-V curves of the membrane under a 50-fold concentration (0.5 M / 10 mM) using different pH electrolytes.

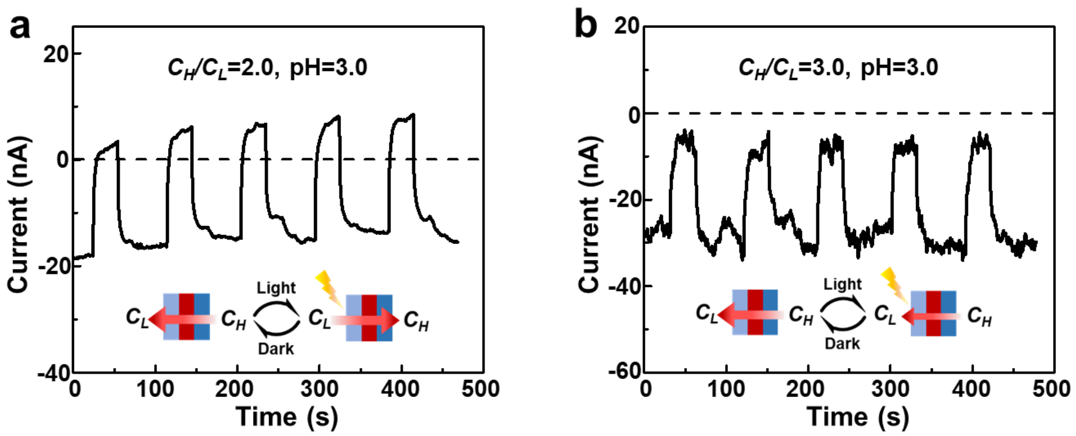

Supplementary Fig. 40 The time-dependent photocurrent cycle of the *p*-BCP@4TPP membrane under (a) 10 mM / 20 mM and (b) 10 mM/ 30 mM concentration gradient (pH=3.0) with a light density of 100.8 mW·cm^{-2}.

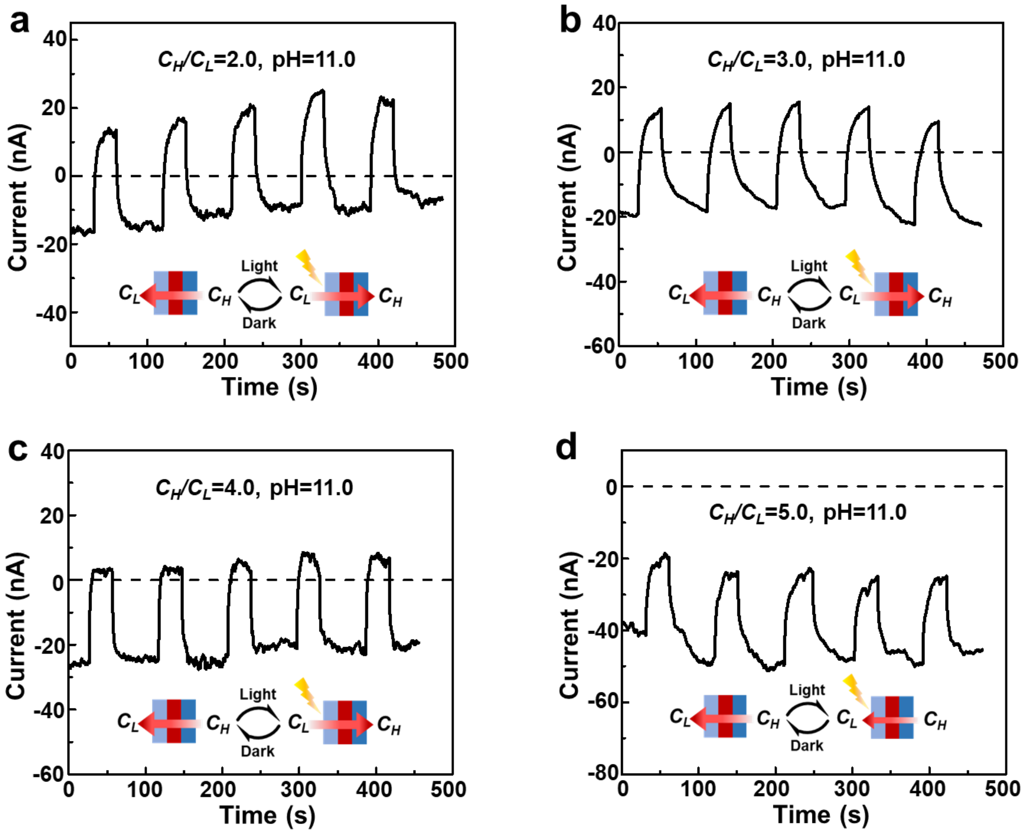

Supplementary Fig. 41 The time-dependent photocurrent cycle of the *p*-BCP@4TPP membrane under (a) 10 mM / 20 mM, (b) 10 mM/ 30 mM, (c) 10 mM/ 40 mM, and (d) 10 mM/ 50 mM concentration gradient (pH=11.0) with a light density of 100.8 mW·cm^{-2}.

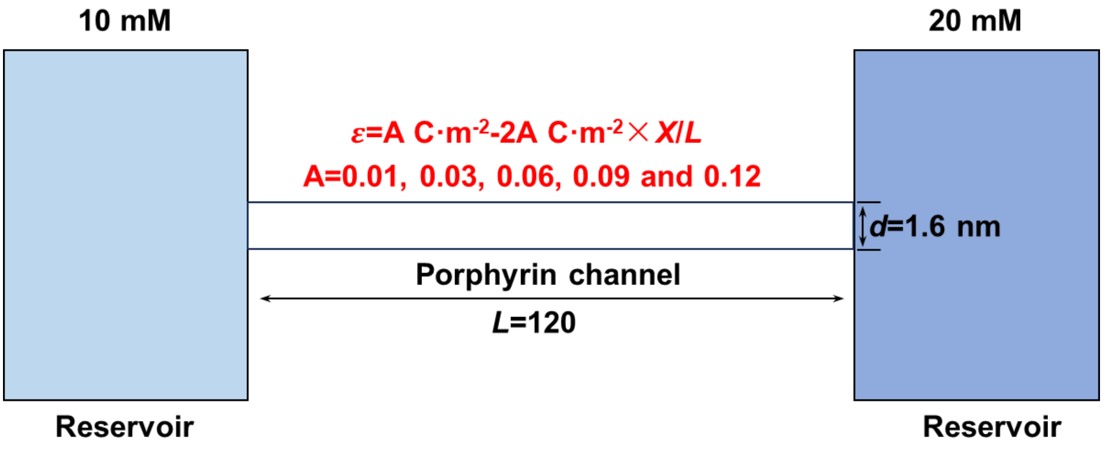

Supplementary Fig. 42 Calculation of porphyrin channel model (not in scale). The theoretical simulation is based on the coupled two-dimensional Poisson-Nernst-Planck equations.

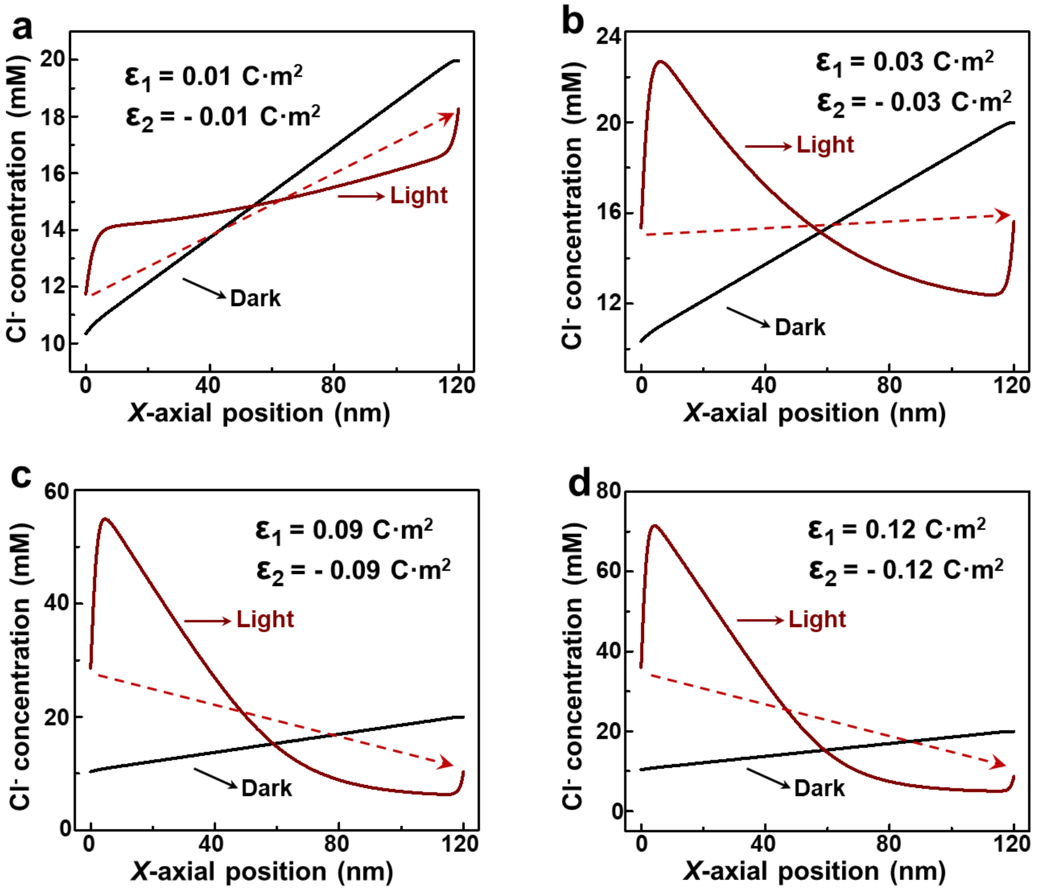

Supplementary Fig. 43 The calculated Cl^- concentration distribution profiles along the X-axis of a single porphyrin channel under different surface charge densities (a-d). The inversed Cl^- concentration distribution indicates the inversed ion migration direction with light irradiation.

Supplementary Tables

Supplementary Table 1. The d-spacing between adjacent porphyrins for all samples

Samples	Q(Å^{-1})^{WAXD}	*d*-spacing (Å)
p-BCP	1.385	4.53
p-BCP@2TPP	1.404	4.47
p-BCP@4TPP	1.432	4.39
p-BCP@8TPP	1.428	4.43

Supplementary Reference

1. Rollings, R. C., Kuan, A. T. & Golovchenko, J. A. Ion selectivity of graphene nanopores. Nat. Commun. 7, 1-7 (2016).

2. Frisch, M. et al. Gaussian 16 Rev. B. 01, Wallingford, CT. 2016.

3. Becke, A. D. Density-functional exchange-energy approximation with correct asymptotic behavior. *Phys. Rev. A* ***38***, 3098-3100 (1988).

4. Becke, A. D. A New Inhomogeneity Parameter in Density-Functional Theory. *J. Chem. Phys.* **109,** 2092-2098 (1998).

5. Lee, C., Yang, W. & Parr, R. G. Development of the colle-salvetti correlation-energy formula into a functional of the electron density. *Phys. Rev. B* **37**, 785-789 (1988).

6. Grimme, S., Antony, J., Ehrlich, S. & Krieg, H. A consistent and accurate *ab initio* parametrization of density functional dispersion correction (DFT-D) for the 94 elements H-Pu. *J. Chem. Phys.* **132**, 154104 (2010).
