# Supplementary material for: Bioinspired light-driven chloride pump with helical porphyrin channels
Source: Nat Commun. 2024 Jan 27;15:832. doi: 10.1038/s41467-024-45117-1 (PMC10821862; doi:10.1038/s41467-024-45117-1)
Supplement: Supplementary file 3 — Description of Additional Supplementary Files [file 41467_2024_45117_MOESM3_ESM.pdf]

Description of Additional Supplementary Files

File Name: Supplementary Data 1
Description: Cartesian coordinates (in Å) of related structures which calculated at the B3LYP-D/6- 31G level of theory.
